# A detailed database of sub-annual Spanish demographic statistics: 2005–2021

**DOI:** 10.1038/s41597-024-02935-8

**Published:** 2024-01-16

**Authors:** Josep Lledó, Jose M. Pavía

**Affiliations:** Department of Applied Economics, Quantitative Methods Area, Universitat de València, Valencia, Spain

**Keywords:** Society, Interdisciplinary studies

## Abstract

The big data revolution has made it possible to collect, transmit and exploit huge amounts of data. The potential this offer for data analysis, however, clashes with the limitations imposed by laws on protection of personal data. This paper details a new database (DEMOSPA0521) made after processing and summarising more than 868 million demographic records from Spain, corresponding to a period of seventeen years (2005–2021). DEMOSPA0521 is composed of fifteen files: a group of (monthly and daily moving averages) datasets derived from population stocks and a collection of (daily, monthly and quarterly) datasets obtained from population, death, migration and birth statistics. The intra-annual distributions were calculated by exploiting both the temporal dimensions of age and calendar. DEMOSPA0521 also includes eleven R-Code files that enables the summary datasets to be derived from the raw microdata. DEMOSPA0521 can be used to confirm established results and employed to answer new research questions.

## Background & Summary

The use of data is crucial for validating, rejecting or modifying hypotheses, as well as instrumental in establishing or anticipating original results. In this regard, the field of demographic research is no exception and routinely uses surveys, administrative datasets or contextual databases to draw conclusions on the behaviour of human populations. The explosion of big data has made it possible to collect, transmit and exploit huge amounts of data, the potential use of which offers many possibilities. However, the wider use of data clashes with the restrictions and limitations imposed by personal data protection laws^1^.

This may explain why, in an oversimplification to avoid exposing personal data, the default databases offered by official bodies that produce demographic information (e.g., in France, Insee, the National Institute of Statistics and Economic Studies; in Spain, INE, the Spanish National Institute of Statistics; or in UK, ONS, the Office for National Statistics) group data together according to a number of variables (such as age, sex or administrative entity), aggregated by annual periods in the two temporal dimensions of age and calendar.

In fact, when gathering demographic data, the norm is to deliver them on an annual basis. For example, the Human Mortality Database^2^, which provides detailed mortality and population data for more than 40 countries and, for the vast majority of countries, over a time horizon of more than 100 years, offers stocks (population) or flows (births and deaths) of demographic statistics (in age intervals and by sex) structured in annual or multi-annual periods. The same is true of other organisations, such as the Wisconsin Longitudinal Study (https://researchers.wls.wisc.edu/) or the Panel Study of Income Dynamics (https://psidonline.isr.umich.edu/), both of which offer demographic and economic data on an annual basis.

The provision of data on an annual basis simplifies the study of temporal evolution of life expectancy or mortality rates, especially in the study of longevity^3,4^. However, it makes it impossible to analyse other phenomena that are also of interest, such as the seasonality of births and migrations or the intra-annual age-calendar patterns of deaths^5,6^.

Driven by our own research, and through exploiting millions of microdata subject to statistical secrecy, we have built new variables and datasets that, while respecting the restrictions imposed by legislation, open up new opportunities for the study of demographic phenomena. This paper details a rich new database (named DEMOSPA0521) that includes stocks (population) and demographic statistical flows (population, deaths, births and migrations) with sub-annual periodicities (quarterly, monthly and weekly and even, in some cases, daily) that, in addition to being used to answer our own research questions, could be reused by other academics to address new areas of research.

The database, created after processing and summarising more than 868 million units of microdata from Spain, corresponding to a period of seventeen years (2005–2021), is composed of two sets of datasets. A group of (monthly and daily moving averages) datasets linked to population stocks and a collection of (daily, monthly and quarterly) datasets associated with population, death, migration and birth statistics. For example, the database contains, on a quarterly basis, the total time exposed to risk contributed by each age group and sex of residents, deceased, migrants or those born in Spain from 2005 to 2021. DEMOSPA0521 is deposited in a non-commercial repository with 10.3886/E192045V2. DEMOSPA0521 opens up new possibilities for future research, including performing deeper analyses of birth seasonality patterns, studying how labour and academic issues impact on migration flows or estimating age-calendar quarterly death rates. In the remainder of this section, these examples are further developed and other potential uses of DEMOSPA0521 presented.

In Spain, the distribution of births within the year has undergone significant changes over time. Between the 1930s and 1960s, there was a noticeable concentration of births in the early months of the year compatible with the hypothesis of a fertility governed primarily by natural factors—concentration of conceptions in spring (births in winter) favoured by the impact of temperature on human fertility^7^. Since then, intra-annual patterns have changed to become consistent with those of a society that exercises effective control of fertility^8^. Initially, there was a shift towards a more uniform distribution of births, with a peak in January due to over-migration with an official birth date of 1 January^9^, and, in more recent years, it appears that people are not only controlling conceptions but also planning them. These shifts prompt a deeper analysis of birth seasonality.

Migratory patterns, both for emigrants and immigrants, exhibit seasonally varying trends, characterized by significant peaks^10^. Therefore, an intriguing avenue for research would be to investigate whether post-summer immigration flows can be attributed to factors such as labour market dynamics or the start of the academic year. Understanding the reasons behind these patterns and their potential consequences, whether in the economic sector or within health and social organisations, warrants further exploration.

DEMOSPA0521 also provides flow statistics in both age and calendar dimensions. The quarterly event-count datasets divide annual flows into sixteen values (see “_3” files in Data Records Section), facilitating the examination of interaction effects between ageing (Q1, Q2, Q3, and Q4) and calendar/seasonal effects (winter, spring, summer, and autumn). Hence, a study to investigate the concentration of deaths in winter and its potential interaction with fractional ages could be performed. A study of this kind could be conducted considering gender and age groups.

A priori, the day on which childbirth occurs is a natural event, governed by chance, so its distribution over the week should be uniform. Its programming and even its induction, however, are increasingly frequent in Western societies^11,12^. Although the weekly distribution of births was thought to be uniform until the 1970s, since then the number of births on weekends has gradually decreased. This result may be a reflection, among other things, of social changes and medical advances registered in the West during recent decades. The data offered in DEMOSPA0521 allow the possibility to reinforce or expand this hypothesis.

Obviously, the studies proposed above are just a few of the possibilities offered by DEMOSPA0521 and other analyses could also be undertaken. Daily statistics could be utilised to analyse the effect of particular holidays such as Christmas or Easter holidays on, for instance, conceptions and migrations, or the data from 2020 in the different datasets could be employed to study the effect of COVID-19 on the different demographic variates. For instance, the set of quarterly datasets (files “D_3” and “_4”) could be used to estimate age-calendar quarterly death rates and seasonal-ageing indexes^6^ and be used to answer the question of whether COVID-19 had an impact on them.

## Methods

The microdata used to build DEMOSPA0521 were acquired through ad-hoc requests and prior payment to the National Statistics Institute (INE) of Spain and included individual records of population stocks^13^, migratory flows^14^ and natural population variations (deaths and births)^15^. These data are gathered by INE from official administrative files, as all bodies (e.g., civil registers) and institutions (e.g., town halls) in Spain are requested by law to share information with INE and to notify any relevant demographic change involving residents in Spain. The microdata were provided under a special agreement contract, according to which under no circumstances were the data to be distributed to third parties. One of the main limitations is that these data are subject to strict conditions of confidentiality and data wrangling. It is forbidden to show individual records or perform crosses between individual records.

To obtain the data, users must submit an official request to the INE via the provided form at https://www.ine.es/infoine/. Prior to data acquisition, a comprehensive application form must be completed, addressing six key points: research entity details, research objectives, requested data, statistical analysis results, data security measures, and information about the research team accessing the data. The request must be signed by the project leader and explicitly approved by the INE.

The records of population stocks are referenced on January 1 and contain, for each year, approximately 46–47 million rows and a total of 5 columns. Each row corresponds to a person residing in Spain on January 1 of each year and the columns report the date of birth (ddmmyyyy), the autonomous community or country of birth, the size of the municipality grouped into six categories^8^, sex and census section of residence; a total of more than 838 million records. As population stocks are referenced on January 1, DEMOSPA0521 is constructed dealing with data from one year longer than the other demographic variables: from 2005 to 2022.

The records of migratory flows come from the statistics of residential variations. In line with the population stocks, the rows of these files correspond to each of the national and international residential variations registered in Spain in each year of study. The columns contain a total of 12 variables. The variables MUNI3 and MUNI2 identify the municipalities (or the country, for an external variation) of destination and origin, respectively. Each record also includes the date of birth (ddmmyyyy) of the migrant and his/her date of change of (country of) residence (ddmmyyyy). Of the other variables, the most relevant are the municipality (or country) of birth, and sex. In these files, a total of 43.3 million records were processed, of which 28.3 and 10.2 million correspond to internal and external emigrants and immigrants, respectively. Part of this data is currently available for free download at the link <https://go.uv.es/OveWpI1>. However, the free files do not report the exact dates of birth or residential variation.

Microdata on deaths and births are part of the vital statistics datasets. For both types of events, the files supplied by INE on payment in advance, in addition to basic variables (such as sex or province), contain detailed information on the place (municipality, district or census section) and the exact moment (in ddmmyyyy format) of occurrence of the event. In the case of deaths, the records also include the exact date (ddmmyyyy) of birth of the deceased. In total, the death and birth files contain 36 and 103 columns, respectively. The total number of records analysed from these statistics amounts to 6.9 (7.3) million deaths (births). Data with less detail (for example, with neither census section nor exact days of birth or death) are available for free download at the links <https://links.uv.es/Gyxm7sP> (deaths) and <https://links.uv.es/bGywq6H> (births).

Compared to the free files, the datasets described above have one characteristic in common: they all have the exact dates (day, month and year) of births and the occurrence of demographic events. This information allows each event to be located on a Cartesian plane of age and calendar, called the Lexis scheme^10^, from which it is possible to build interesting summary statistics. From this representation, for example, the intra-annual distributions of the main demographic variables can be calculated.

The new generated variables are organised within DEMOSPA0521 in 15 files/datasets, whose names follow a specific coding that makes it easy to locate and identify their content. The first character of the name of each file reports the type and origin of the basic statistic: P, E, I, D and B to denote Population, Emigrants, Immigrants, Deaths and Births, respectively. The second relevant character, of numeric type, corresponds to the explanatory table number of this document. A small descriptive text is added to these two codes. For example, the file “*P_1_Number_of_people_per_month_of_birth*” contains the result of the calculation, based on the population microdata (P), of the number of people (by sex) residing in Spain by month of birth on January 1 of each reference year. The data of all the tables are offered in plain text files, with ‘csv’ extensions, data entries delimited with semicolons (;), points as decimal separators and *long table* structures. This facilitates its subsequent treatment in the main free software packages: dplyr (R language) or Pandas (Python language). The R code created to generate each file adheres to the same nomenclature as the final files.

**Fig. 1:**
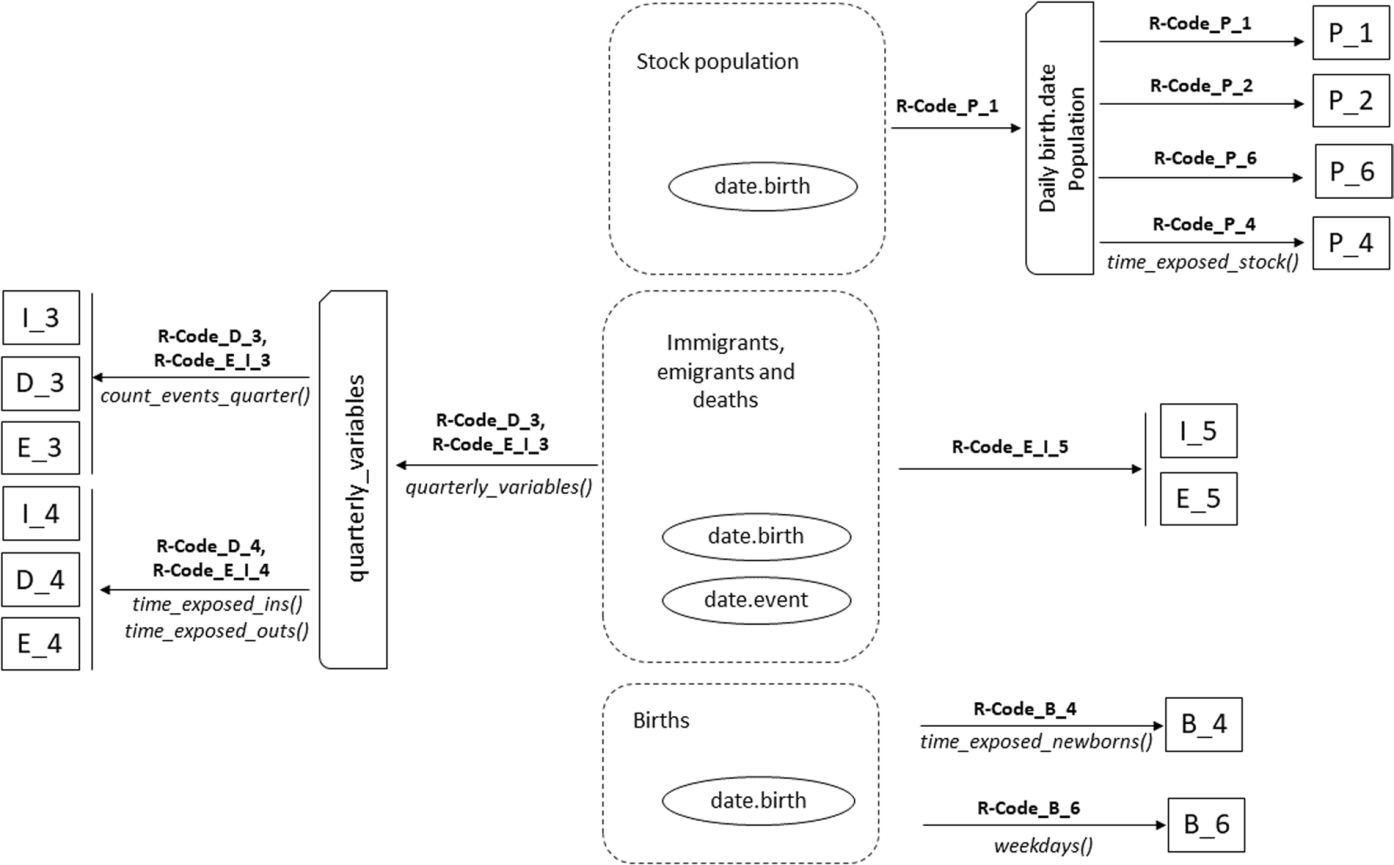
Schematic overview of the workflow for creating the DEMOSPA0521. This map illustrates the connections between microdata variables (date.birth or date.event, represented by ellipsis) and the final files in a rectangular format. The files are generated using R functions, primarily sourced from the *qlifetable* package^17^. Additionally, intermediate files, marked by a rectangle with one corner cut off, are necessary in the generation process. The map also details the R-format files (encoded as R-Code) also deposited in DEMOSPA0521 that facilitate the reproduction of the calculations.

To generate the files, the content of which is described in the following sections, more than 868 million records were processed individually. All the data processing described and presented in this paper was performed using ad-hoc scripts in the statistical software R, version 4.2.1^16^. Much of the datasets presented in this paper were obtained by processing the microdata using the R package *qlifetable*^17^. Every final file has been acquired based on the specified scheme detailed in Fig. 1.

Figure 1 illustrates that the microdata in date format, denoted by ellipses, serve as the origin for all files in DEMOSPA0521. The dates of births (denoted as date.birth) are mandatory for summarizing various demographic events, including stock population, migrations, deaths, and births. Meanwhile, dates of events (denoted as *date.event*) play a crucial role in analysing demographic trends that affect population change, such as migrations and deaths.

Files P_1, P_2, P_4 and P_6 (denoted by a rectangle) are established by acquiring an intermediary file (*daily birth.date population*, denoted by a rectangle with one corner cut off) that includes the count of individuals born on each day (in ddmmyyyy format). Unfortunately, the provision of this file poses a challenge, particularly for certain dates, mainly in adult age groups, where only one person may be alive. Consequently, presenting disaggregated values for such dates is not possible. Should the user possess the birth dates of the population stock, the *daily birth.date population* file could be generated for birth dates of population using the code in R-Code_P_1. This not-provided intermediate file structure allows the files P_1, P_2, P_4 and P_6 to be obtained. The *time_exposed_stock*() function in the *qlifetable* package is used to obtain the P_4 file. Furthermore, R-Code_P_1, R-Code_P_2, R-Code_P_4 and R-Code_P_6 are employed to acquire P_1, P_2, P_4 and P_6, respectively.

To derive the count of events for each quarterly age and calendar relationship (D_3, E_3, and I_3), microdata involving dates of births and events serve as the foundation. Initially, the function *quarterly_variables()* from the *qlifetable* package is applied to obtain the intermediate file (quartely_variables). This step facilitates the computation of precise risk coordinates in the Lexis diagram and quarterly biometric variables. Subsequently, the *count_event()* function is employed on these intermediate files to generate D_3, E_3, and I_3. All the requisite code is accessible in the R-Code_D_3 and R-Code_E_I_3 files.

The D_4, E_4, and I_4 block file enables both exposure and non-exposure times to be obtained for each of the three demographic events. To acquire these, the functions *time_exposed_ins()* and *time_exposed_outs()* from the qlifetable package are employed from the intermediate quarterly_variables file. The complete process is referenced in the scripts R-Code_D_4 and R-Code_E_I_4. Files E_5 and I_5 represent the outcome of computing the daily moving average, with *k* = 3, for emigrants and immigrants using the file R-Code_E_I_5. Finally, the births section comprises two files. The first, B_4, is produced by the R-Code_B_4, employing the main function *time_exposed_newborns()* function from the *qlifetable* package. The second, B_6, is generated by R-Code_B_6, utilizing the *weekdays()* function from R base. The methodology for obtaining sub-annual times of exposure is extensively developed in studies by Pavía and Lledó^6^.

## Data Records

The datasets and the R-Code are available at openICPSR in 10.3886/E192045V2^18^. Usually, the detailed data of stocks or population censuses offered are *referenced/linked* to a time instant (for example, the beginning of each year) and a territory, aggregated by age groups or individual ages and sex (see, for instance, Insee and INE). From the exploitation of microdata, DEMOSPA0521 offers more detailed population stocks.

The file (dataset) called “*P_1_Number_of_people_per_month_of_birth.csv”* reports on the number of people residing in Spain on the date indicated in *valuation_date* by sex, age and month of birth. The *gender*, *birth_year* and *birth_month* columns indicate the sex, year and month of birth of the group of people whose magnitude is reported in *number_of_persons*. The data offered in P_1 do not exploit the full potential offered by the dates of the population microdata. From these we could calculate the total number of people born on each birthday (ddmmyyyy) for each *valuation_date*. Unfortunately, this level of detail cannot be included in DEMOSPA0521 since it would violate the conditions stipulated in the data transfer. The exact date of each birth, however, is somewhat random, as it could reasonably have been a few days earlier or later. Information equivalent to that which cannot be offered, therefore, could be contained in a dataset built under the previous premise, which can be shown. Specifically, if we define *t* as the birthday, we can compute the k-order moving average of the number of people, *P*_*t*_, born on day *t* as $M{A}_{t}\left(k\right)=\frac{1}{2k+1}{\sum }_{j=0}^{2k}{P}_{t-k+j}$. The data offered in two files “*P_2_Moving average (k* = 3*) per day of birth men.csv*” for men and “*P_2_Moving average (k* = 3*) per day of birth women.csv*” for women is obtained for *k* = 3. Since the number of people living to very old ages is very low, P_2 files do not contain statistics for dates before 100 years of the valuation date to safeguard the confidentiality of the individual records. In any case, once the microdata have been acquired this restriction can be removed by running the code present in R-Code_P_2.

Demographic flow statistics count, among other variables, the number of natural and migratory events that are registered in a population during a period of time. Having sub-annual statistics of these variables is essential for a more complete study of demographic phenomena. Seasonal patterns, especially in deaths, have been extensively studied from the demographic, epidemiological and sociological point of views^5,19^, although their interaction with age has received less attention despite the fact that several studies^6,10,20^ show that intra-age and calendar patterns have a not-innocuous impact on the actuarial calculation. DEMOSPA0521 offers a collection of datasets that allows the sub-annual patterns of the flow demographic statistics to be studied in the two dimensions, age and calendar, and in their interaction.

A first group of datasets is composed by the files “*D_3_Count_events_by_quarter.csv*”, “*E_3_Count_events_by_quarter.csv*” and “*I_3_Count_events_by_quarter.csv*”, containing respectively the number of deaths, emigrants and immigrants (number_events) registered in Spain by sex (*gender*) for each combination of age (quarter_age) and calendar quarter (quarter_calendar) in each year (valuation_year). In these files, age quarters are identified by Q1, Q2, Q3 and Q4 and calendar quarters (seasons) by Winter (January, February and March), Spring (April, May and June), Summer (July, August and September) and Autumn (October, November and December). Note that the correspondence between months and actual (either climatological or astronomical) seasons is approximate.

In addition to the number of events recorded in each age-calendar quarter, DEMOSPA0521 also offers, with the same structure, the total time exposed to the risk of death associated with each sex- and age-group of deaths, migrants and new-borns as well as the expected time exposed of the stocks of populations as of January 1, assuming that all the residents survive the whole year. This information is offered in the csv files D_4, E_4, I_4, B_4 and P_4, which include the text “*Time_exposed_by_quarter*” as explanatory text in their name.

The time-exposed statistics, however, are not enough to calculate the total time of exposure corresponding to each combination of age-calendar quarter, integer-age and year. To do this, we also need to have the time of non-exposure of emigrants and deaths. These figures, together with the equivalent times for immigrants, are also available in the datasets D_4, E_4 and I_4. Using these statistics, one can easily compute the total time of exposure in each quarter by (i) summing (by age-calendar quarter, integer-age and year) the time exposed recorded in P_4, I_4 and B_4 and (ii) subtracting the time not-exposed recorded in D_4 and E_4.

The “_3” and “_4” files summarise flows on a quarterly basis. DEMOSPA0521 also contains daily/weekly syntheses of flow demographic variates. The files I_5 and E_5, grouped in three age groups ([0–16), [16–65), >65), offer daily moving averages (*k* = 3) for the number of immigrants and emigrants registered in Spain during 2005–2021, respectively. The data in I_5 and E_5 allow us to study the seasonality of foreign demographic flows in greater detail.

Finally, the files B_6 and P_6 offer statistical summaries of dates of birth grouped by day of the week. B_6 contains the number of births registered in Spain by sex, day of the week and *valuation_year* (2005–2021) and P_6 accounts for the weekday distributions of birthdays of the stocks of populations.

## Technical Validation

In this study, we have obtained, stored, processed, and summarized over 868 million microdata. Throughout this process, we have implemented various data validation controls. Specifically, we have conducted a validation check to ensure the accuracy of the total time of exposure to risk and non-exposure to risk for deaths (see Table 1), emigrants (see Table 2), and immigrants (see Table 3). This validation was performed by comparing the data in the “*4_Time_exposed_by_quarter.csv*” file, combining the exposure time with the non-exposure time, with the number of individuals recorded in the “*3_Count_events_by_quarter.csv* “ file. The matching of the two files (_3 and _4) exhibited minimal discrepancies. The small differences are due to the age of 0 years since a baby who, for example, dies at less than one year of age has been counted with a value of 1 in D_3 while the sum of its exposure and non-exposure times are, in this case, less than one.

**Table 1:** Relative discrepancies between number of death events and exposure times by year and sex.

Year	Men			Women		
	D_3	D_4	Differences	D_3	D_4	Differences
**2005**	201,769	201,349	−0.21%	185,586	185,287	−0.16%
**2006**	194,154	193,755	−0.21%	177,324	177,031	−0.16%
**2007**	201,136	200,740	−0.20%	184,225	183,905	−0.17%
**2008**	199,647	199,247	−0.20%	186,677	186,371	−0.16%
**2009**	199,095	198,748	−0.17%	185,838	185,558	−0.15%
**2010**	198,121	197,789	−0.17%	183,926	183,626	−0.16%
**2011**	199,854	199,528	−0.16%	188,057	187,769	−0.15%
**2012**	205,920	205,603	−0.15%	197,030	196,764	−0.14%
**2013**	199,834	199,572	−0.13%	190,585	190,372	−0.11%
**2014**	201,571	201,272	−0.15%	194,259	194,020	−0.12%
**2015**	213,309	213,043	−0.12%	209,259	209,062	−0.09%
**2016**	208,993	208,719	−0.13%	201,618	201,430	−0.09%
**2017**	214,236	213,977	−0.12%	210,287	210,085	−0.10%
**2018**	216,442	216,208	−0.11%	211,279	211,098	−0.09%
**2019**	212,683	212,459	−0.11%	206,020	205,840	−0.09%
**2020**	249,664	249,469	−0.08%	244,112	243,965	−0.06%
**2021**	231,410	231,204	−0.09%	219,334	219,167	−0.08%

The column *differences* shows the relative discrepancies between the columns *number_events* in the file “D_3_count_events_quarter.csv” and the columns *time_exposed* and *time_not_exposed* in the file “D_4_Time_exposed.csv”, aggregated in column D_4.

**Table 2:** Relative discrepancies between number of emigration events and exposure times by year and sex.

Year	Men			Women		
	E_3	E_4	Differences	E_3	E_4	Differences
**2005**	38,246	38,221	−0.06%	29,765	29,740	−0.08%
**2006**	82,151	82,112	−0.05%	60,145	60,114	−0.05%
**2007**	132,848	132,795	−0.04%	94,217	94,172	−0.05%
**2008**	153,475	153,414	−0.04%	112,985	112,922	−0.06%
**2009**	188,756	188,685	−0.04%	134,885	134,821	−0.05%
**2010**	218,221	218,164	−0.03%	155,733	155,682	−0.03%
**2011**	211,620	211,565	−0.03%	158,920	158,867	−0.03%
**2012**	217,348	217,286	−0.03%	159,701	159,641	−0.04%
**2013**	255,000	254,945	−0.02%	198,542	198,492	−0.03%
**2014**	245,303	245,252	−0.02%	200,264	200,217	−0.02%
**2015**	232,990	232,948	−0.02%	194,152	194,110	−0.02%
**2016**	224,723	224,674	−0.02%	186,831	186,790	−0.02%
**2017**	222,765	222,721	−0.02%	188,479	188,438	−0.02%
**2018**	186,922	186,880	−0.02%	161,128	161,093	−0.02%
**2019**	184,601	184,562	−0.02%	161,148	161,115	−0.02%
**2020**	145,650	145,627	−0.02%	125,575	125,547	−0.02%
**2021**	244,719	244,692	−0.01%	207,792	207,771	−0.01%

The column *differences* show the relative discrepancies between the columns *number_events* in the file “E_3_count_events_quarter.csv” and the columns *time_exposed* and *time_not_exposed* in the file “E_4_Time_exposed.csv”, aggregated in column E_4.

**Table 3:** Relative discrepancies between number of immigration events and exposure times by year and sex.

Year	Men			Women		
	I_3	I_4	Differences	I_3	I_4	Differences
**2005**	388,822	388,344	−0.12%	330,462	330,017	−0.13%
**2006**	441,825	441,233	−0.13%	399,019	398,469	−0.14%
**2007**	520,911	520,261	−0.12%	437,355	436,715	−0.15%
**2008**	387,308	386,648	−0.17%	338,701	338,122	−0.17%
**2009**	253,881	253,280	−0.24%	245,096	244,511	−0.24%
**2010**	238,931	238,343	−0.25%	225,512	224,967	−0.24%
**2011**	230,862	230,251	−0.26%	223,824	223,296	−0.24%
**2012**	189,387	188,888	−0.26%	181,126	180,661	−0.26%
**2013**	176,521	176,033	−0.28%	165,869	165,410	−0.28%
**2014**	205,041	204,545	−0.24%	194,903	194,413	−0.25%
**2015**	233,702	233,148	−0.24%	221,976	221,452	−0.24%
**2016**	269,407	268,800	−0.23%	265,167	264,612	−0.21%
**2017**	320,929	320,253	−0.21%	316,446	315,858	−0.19%
**2018**	387,085	386,421	−0.17%	373,719	373,122	−0.16%
**2019**	447,009	446,288	−0.16%	426,833	426,171	−0.16%
**2020**	273,969	273,492	−0.17%	249,649	249,227	−0.17%
**2021**	348,943	348,405	−0.15%	313,230	312,736	−0.16%

The column *differences* show the relative discrepancies between the columns *number_events* in the file “I_3_count_events_quarter.csv” and the columns *time_exposed* and *time_not_exposed* in the file “I_4_Time_exposed.csv”, aggregated in column I_4.

Another significant validation was conducted regarding population stocks and involves the files “*P_1_Number of people per month of birth*”, “*P_4_Time_exposed_by_quarter*” and “*P_6_Births per day of the week of population*” (see Table 4). These files record the number of individuals born per month, the number of individuals categorized by age and calendar quarter, and the number of individuals grouped by the day of the week of birth. During this validation process, very narrow differences were observed in 2005 and 2006, coinciding in the rest of the years (see Table 4). It is important to note that these insignificant differences are solely attributable to the implementation of measures to ensure the anonymity of the individuals involved.

**Table 4:** Aggregation of population stocks by year and sex after being summarized using different statistical transformations.

Year	Men			Women		
	P_1	P_4	P_6	P_1	P_4	P_6
**2005**	21,780,869	21,780,861	21,780,866	22,327,661	22,327,656	22,327,660
**2006**	22,100,466	22,100,460	22,100,466	22,608,498	22,608,495	22,608,498
**2007**	22,339,962	22,339,962	22,339,962	22,860,775	22,860,775	22,860,775
**2008**	22,847,737	22,847,737	22,847,737	23,310,085	23,310,085	23,310,085
**2009**	23,116,988	23,116,988	23,116,988	23,628,819	23,628,819	23,628,819
**2010**	23,226,185	23,226,185	23,226,185	23,794,846	23,794,846	23,794,846
**2011**	23,283,187	23,283,187	23,283,187	23,907,306	23,907,306	23,907,306
**2012**	23,298,356	23,298,356	23,298,356	23,966,965	23,966,965	23,966,965
**2013**	23,196,386	23,196,386	23,196,386	23,933,397	23,933,397	23,933,397
**2014**	22,985,676	22,985,676	22,985,676	23,785,665	23,785,665	23,785,665
**2015**	22,890,383	22,890,383	22,890,383	23,733,999	23,733,999	23,733,999
**2016**	22,843,610	22,843,610	22,843,610	23,713,398	23,713,398	23,713,398
**2017**	22,832,861	22,832,861	22,832,861	23,739,271	23,739,271	23,739,271
**2018**	22,896,602	22,896,602	22,896,602	23,826,378	23,826,378	23,826,378
**2019**	23,042,428	23,042,428	23,042,428	23,983,780	23,983,780	23,983,780
**2020**	23,255,590	23,255,590	23,255,590	24,195,205	24,195,205	24,195,205
**2021**	23,222,953	23,222,953	23,222,953	24,162,154	24,162,154	24,162,154

Columns P_1, P_2 and P_3 are aggregations by year and sex of the columns *number_of_persons*, *time_exposed* and *number_of_birthdays* as available in, respectively, the files “P_1_Number of people per month of birth.csv”, “P_4_Time_exposed_by_quarter.csv” and “P_6_Births per day of the week of population.csv”.

## Usage Notes

The utilization of data comes with inherent limitations, in part derived from the underlying quality of the raw microdata. In this sense, our microdata are also exposed to some of the limitations highlighted by Cairns’ study^21^. In their paper ‘Phantoms never die: living with unreliable population data’, Cairns and colleagues analyse national mortality trends by examining the quality of data related to population, exposure, and deaths in England and Wales. Their primary findings reveal significant disparities in births of the 1919 and 1947 cohorts^21^ and that uneven patterns of births within a given calendar year are a major cause of errors in population and exposure data^21^ when they are used aggregated. Although our datasets do not suffer from this limitation as all our computations consider detailed dates, they are still exposed to anomalies in registering deaths, also identified in the study by Cairns *et al*.

Migration data present a significant challenge due to inconsistencies. Various models^22,23^ have been explored to estimate missing flows. Another source of discrepancy arises from common errors observed on specific dates. Official agents often designate the first day of January as the birth date for immigrants lacking precise birth information. This practice artificially generates peaks in the data^9,10^, which are resolved by randomly distributing the excesses.

Another crucial aspect concerns the timing of foreign migrations. The temporal alignment of information with emigration/immigration events, as reflected in the Residential Variations statistics (EVR), relies on a local administrative process. It is important to note that this timing is approximate and may not accurately represent the real-time occurrence of some migratory events. In the context of foreign emigrations involving non-EU immigrants, the emigration date is inferred from an administrative procedure known as ‘Bajas por Caducidad’ (Deregistration due to expiration). This procedure may introduce a delay of up to 2 years from the actual emigration date, further emphasizing the potential divergence from real-time events.

Finally, it is important to note that the data under consideration are not exempt from what are commonly referred to as transcription errors, a typical occurrence in datasets of this nature. Additionally, one must be vigilant regarding inaccuracies in recording dates, as well as the frequent occurrence of delayed registration for certain demographic events, such as births. As highlighted in the report^24^, these challenges contribute to the complexity of ensuring the accuracy and reliability of the data in question.

## Data Availability

The data analysis methods, software and associated parameters used in this study are described in the section of Methods. The code used to derive all datasets from the raw microdata can be downloaded at 10.3886/E192045V2.
